# Exploring Genetic Association of Tea Intake With Allergic Diseases Among European Population: A Bidirectional Mendelian Randomization Study

**DOI:** 10.1002/fsn3.4574

**Published:** 2024-11-01

**Authors:** Jinjin Zhang, Yuhan Liu, Jiawei Zhang, Fei Zeng, Yuqing Wu, Xuexue Zhang, Daying Zhang, Mengye Zhu

**Affiliations:** ^1^ Department of Pain Medicine Ji'an Central People's Hospital Ji'an China; ^2^ Key Laboratory of Neuropathic Pain, Healthcare Commission of Jiangxi Province Nanchang China; ^3^ School of Basic Medical Sciences Jiangxi Medical College, Nanchang University Nanchang China; ^4^ Department of Pain Medicine The First Affiliated Hospital, Jiangxi Medical College, Nanchang University Nanchang China; ^5^ Department of Anesthesiology The Affiliated Stomatological Hospital of Nanchang University Nanchang China

**Keywords:** allergic asthma, allergic diseases, allergic rhinitis, atopic dermatitis, mendelian randomization, tea intake

## Abstract

Previous observational studies focused on the association of tea intake and allergic diseases. However, it is not known whether these associations are causal. We used a bidirectional Mendelian randomization (MR) study to assess the causal relationship of tea intake with the risk of allergic diseases, such as atopic dermatitis (AD), allergic rhinitis (AR), and allergic asthma (AA). Single‐nucleotide polymorphisms (SNPs) which had genetic statistical significance with tea intake were used as instrumental variables (IVs). We employed heritable IVs of tea intake from the UK Biobank, which included 447,485 samples. Sensitivity analyses were further performed using MR Egger and MR‐PRESSO. Inverse variance weighted (IVW) method was used as the main approach. In this MR study, 40 independent SNPs were selected for tea intake. The MR analysis revealed that an increase in genetically predicted tea intake was associated with a lower risk of AD (OR = 0.709, 95% CI = 0.546–0.919, *p* = 0.009). Furthermore, we observed a causal effect of genetically predicted tea intake on the risk of AA (OR = 0.498, 95% CI = 0.320–0.776, *p* = 0.002). However, no significant causal relationship was found between genetically predicted tea intake and AR (OR = 1.008, 95% CI = 0.998–1.017, *p* = 0.115). Our MR analysis suggested that increased tea intake may reduce the risk of AD and AA in European population. This suggests that tea intake is likely a trigger or a prevention strategy for AD and AA.

## Graphical abstract

Our recent study utilizing bidirectional Mendelian randomization from the UK Biobank has indicated that increased tea consumption is significantly associated with reduced risks of atopic dermatitis and allergic asthma, while no significant effect was observed for allergic rhinitis. These results suggest that tea consumption may serve as a preventive strategy for atopic dermatitis and allergic asthma, particularly in the European population. Further exploration of this association could inform dietary recommendations for allergy management.

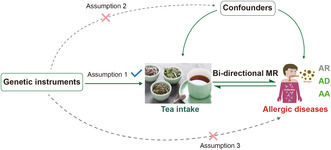

## 1 Introduction

Over the past 3 decades, there has been a significant increase in the prevalence of allergic diseases, such as atopic dermatitis (AD), allergic rhinitis (AR), and allergic asthma (AA), resulting in a substantial societal burden (Nwaru and Virtanen 2017; Platts‐Mills 2015). Atopic dermatitis, a common inflammatory skin condition, is characterized by recurrent eczematous lesions. Allergic rhinitis, a noncontagious inflammation of the nasal mucosa, presents with symptoms such as intermittent sneezing, nasal discharge, itching, and congestion. Allergic asthma is an inflammatory condition of the airways that manifests as periodic wheezing, chest tightness, shortness of breath, and mucus production. Currently, there is no definitive treatment for AD, AR, or AA. Glucocorticoids and antihistamines are commonly used to manage these allergic conditions; however, symptoms often recur after discontinuation of these drugs (Cook, Argenio, and Lovinsky‐Desir 2021). While AD, AR, and AA are associated with genetic factors, diet, and environmental factors such as air pollution and allergen exposure, the exact underlying causes remain elusive (Boutin et al. 2020). Identifying these potential risk factors is crucial not only for understanding the etiology of these conditions but also for developing more effective prevention and treatment strategies.

Tea, the second most popular beverage after water, is consumed by more than two‐thirds of the world's population (Hemler and Hu 2019). There is an increasing amount of evidence linking tea consumption to a variety of health benefits, including antioxidant (Al‐Awaida et al. 2019), antidiabetic (Fu, Fu, et al. 2017; Fu, Li, et al. 2017), antitumor (Wang et al. 2018), and anti‐β‐amyloid effects (Polito et al. 2018). Furthermore, tea has anti‐allergic properties due to its numerous bioactive compounds such as polyphenols, polysaccharides, and saponins. Recent research suggests that leaves from tea cultivars with a high concentration of methylated catechins are more effective in reducing allergic symptoms (Li et al. 2021).

Mendelian randomization (MR) is a method of employing genetic variants, primarily single‐nucleotide polymorphisms (SNPs), as instrumental variables (IVs) to evaluate the potential causal influence of exposure on outcomes (Burgess et al. 2015; Davey Smith and Hemani 2014). As genetic variations are randomly assigned during meiosis and remain unchanged after fertilization, the MR methodology can circumvent the drawbacks of conventional epidemiologic studies, such as confounding factors, reverse causation, and selection biases (Lawlor et al. 2008). The causal association between tea intake with allergic diseases has not been examined using MR. We hypothesized that the consumption of tea may contribute to a reduction in the risk of allergic diseases within the European population. Consequently, we performed a bidirectional two‐sample MR analysis to ascertain the causation.

## 2 Materials and Methods

### 2.1 Study Design

**FIGURE 1:**
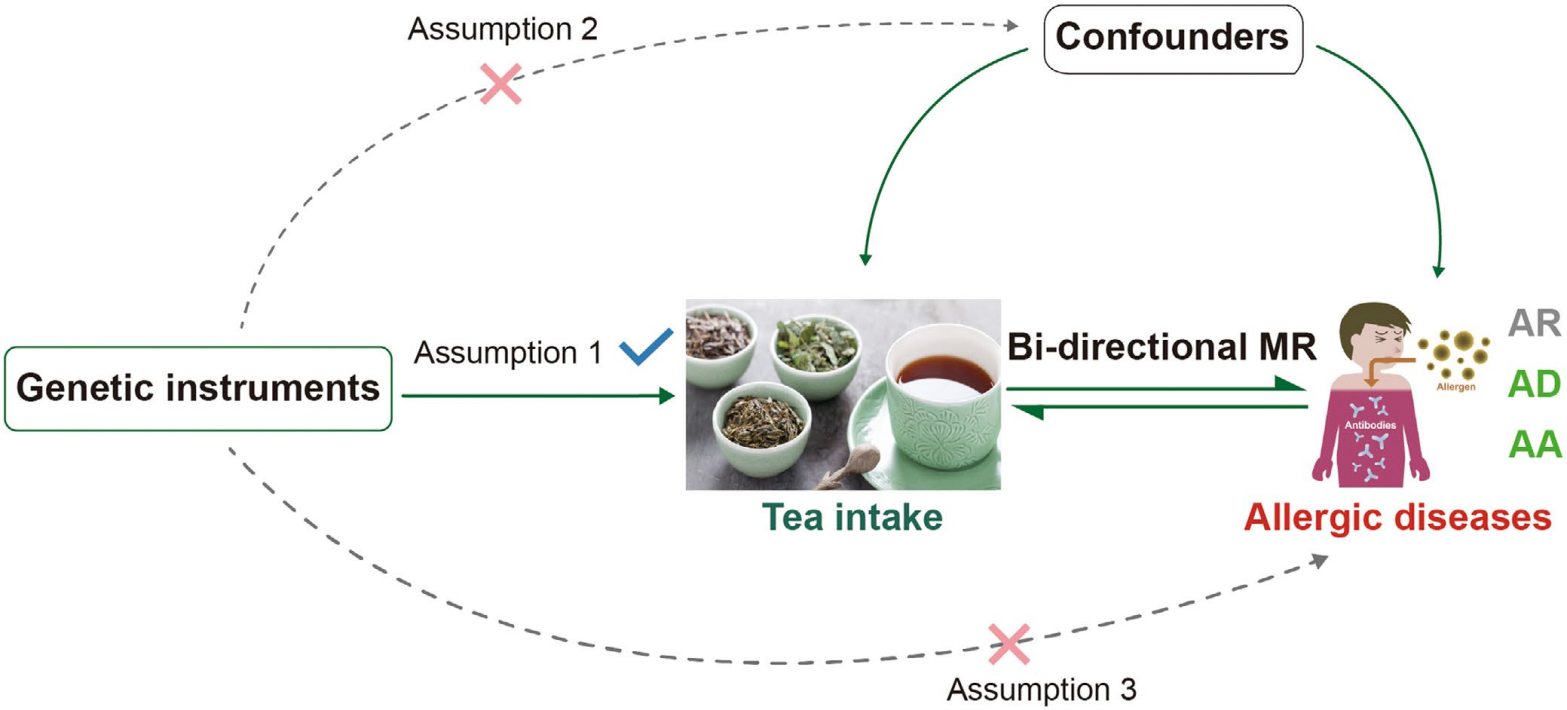
Overview of the study design.

To determine whether there is a correlation between tea intake and allergic diseases (including AD, AR, and AA), we conducted a bidirectional two‐sample MR study using publicly accessible Genome‐Wide Association Studies (GWASs). The MR methodology is predicated on three assumptions: (1) The genetic variants utilized as IVs have a significant association with the exposure; (2) the genetic variants are not linked with any confounding variables; (3) there is no direct relationship between the genetic variation and the outcome, except through the exposure (Glymour, Tchetgen Tchetgen, and Robins 2012). Our MR framework design is depicted in Figure 1.

### 2.2 Data Sources

To conduct the MR analyses, we obtained summary statistics derived from publicly accessible GWAS for each trait. We employed heritable IVs of tea intake from the UK Biobank, which included 447,485 samples. Specifically, the UK Biobank is a cohort study that engaged participants from the UK, aged between 40 and 69 years (Bycroft et al. 2018). Data regarding tea drinking habits were gathered through the questionnaire entry: “How many cups of tea do you drink each day? (Include black and green tea).” Detailed information can be accessed on the IEU website (https://gwas.mrcieu.ac.uk). We derived IVs for AD from the most recent and comprehensive meta‐analysis study. This study encompassed a total of 796,661 individuals of European descent from three biobanks: the Estonian Biobank (with 11,187 cases and 125,537 controls), FinnGen (with 8383 cases and 236,161 controls), and the UK Biobank (with 2904 cases and 412,489 controls) (Sliz et al. 2022). For AR, summary data was extracted from the GWAS dataset of 484,598 individuals, including 27,415 cases and 457,183 controls (Dönertaş et al. 2021). Summary‐level data for AA was extracted from the FinnGen. A total of 4859 cases and 131,051 controls were included in this GWAS. Information on all genetic datasets in this study is shown in Table S1. Table S2 shows the sources of data used to identify genetic variants in this study.

### 2.3 Selection and Validation of SNPs

SNPs associated with tea intake at the genome‐wide significance threshold with *p* < 5 × 10^−8^ were selected. The independence among these chosen SNPs was evaluated based on pairwise linkage disequilibrium (LD). LD across these SNPs was calculated using the 10,000 genomes LD clumping (*r* ^2^ < 0.001) (Machiela and Chanock 2015). To determine the strength of IVs, F statistics (*β* ^2^/SE^2^) (Emdin, Khera, and Kathiresan 2017; Pierce, Ahsan, and Vanderweele 2011; Zhang et al. 2022) were utilized. IVs with an F‐statistic < 10 were considered weak instruments (Burgess et al. 2011). Table S1 displays the IVs that are ultimately used.

### 2.4 Ethical Approval

In our study, all exposure and outcome datasets were derived from publicly available, de‐identified data sources. The original studies from which these datasets were obtained had received prior approval from their respective ethics committees, and informed consent was secured from all participants (Bycroft et al. 2018; Dönertaş et al. 2021). Given that our study utilized only summary‐level data, it was exempt from requiring additional ethical approval.

### 2.5 Statistical Analysis

The inverse variance weighted (IVW) meta‐analysis was considered the primary analysis. To identify and exclude potential pleiotropic instruments, we performed the Mendelian randomization pleiotropy residual sum and outlier test (MR‐PRESSO) (Verbanck et al. 2018). Outlier instrumental variables identified by the MR‐PRESSO analysis were removed incrementally to reduce the effect of horizontal pleiotropy. We also conducted sensitivity analyses using the weighted median and MR Egger methods. The weighted median method can yield valid estimates if more than 50% of the information originates from valid IVs. The MR Egger method can assess the horizontal pleiotropy of selected IVs (Burgess et al. 2017). Cochrane's *Q* value can indicate heterogeneity among selected IVs (Bowden, Davey Smith, and Burgess 2015). Additionally, a leave‐one‐out analysis was performed to investigate whether significant results were influenced by a specific SNP. All statistical analyses were performed using the “TwoSampleMR” and “MRPRESSO” packages in R version 4.2.3.

## 3 Results

### 3.1 Genetically Predicted Tea Intake on the Risk of Allergic Diseases

**FIGURE 2:**
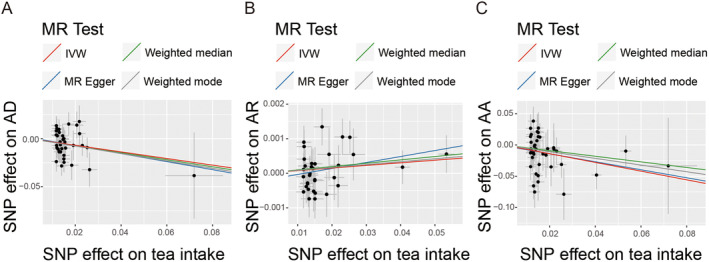
Scatter plots of potential effects of SNPs on tea intake and the risk of allergic diseases. (A) Scatter plot of tea intake‐AD risk MR. (B) Scatter plot of tea intake‐AR risk MR. (C) Scatter plot of tea intake‐AA risk MR. AA, allergic asthma; AD, atopic dermatitis; AR, allergic rhinitis; SNP, single‐nucleotide polymorphism.

The IVs utilized for the MR analyses of tea intake and allergic diseases are detailed in Table S1. In this MR study, 40 independent SNPs were selected for tea intake. The F statistics for all SNPs exceeded the conventional threshold of 10, which is commonly used to distinguish between strong and weak instrument bias. The MR analysis revealed that an increase in genetically predicted tea intake was associated with a lower risk of AD (OR = 0.709, 95% CI = 0.546–0.919, *p* = 0.009), a finding that was corroborated by the weighted median model (Table 1). Furthermore, we observed a causal effect of genetically predicted tea intake on the risk of AA, with an odds ratio of 0.498 (95% CI = 0.320–0.776, *p* = 0.002). However, no significant causal relationship was found between genetically predicted tea intake and AR (OR = 1.008, 95% CI = 0.998–1.017, *p* = 0.115). The estimated effect sizes for SNPs associated with tea intake on AD, AR, and AA were illustrated in a scatter plot (Figure 2).

**TABLE 1:** Odds ratios for the associations between genetically predicted tea intake and allergic diseases.

Outcomes	Cases	Controls	nSNPs	Method	OR (95% CI)	*p*
AD	774,187	796,661	37			
				MR Egger	0.658 (0.260, 1.662)	0.382
				Weighted median	0.694 (0.483, 0.996)	0.048
				IVW	0.709 (0.546, 0.919)	0.009
				Weighted mode	0.680 (0.375, 1.231)	0.211
AR	457,183	484,598	39			
				MR Egger	1.017 (0.996, 1.039)	0.115
				Weighted median	1.010 (0.996, 1.024)	0.164
				IVW	1.008 (0.998, 1.017)	0.115
				Weighted mode	1.008 (0.993, 1.024)	0.303
AA	131,051	135,910	40			
				MR Egger	0.527 (0.197, 1.408)	0.209
				Weighted median	0.638 (0.348, 1.168)	0.145
				IVW	0.498 (0.320, 0.776)	0.002
				Weighted mode	0.586 (0.308, 1.116)	0.112

Abbreviations: CI, confidence interval; IVW, inverse variance weighted; nSNPs, number of single‐nucleotide polymorphisms; OR, odds ratio.

### 3.2 Genetically Predicted Allergic Diseases on the Risk of Tea Intake

**FIGURE 3:**
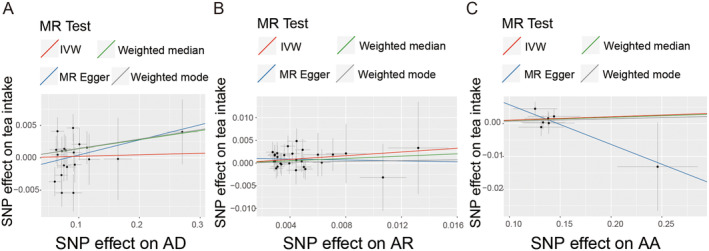
Scatter plots of potential effects of SNPs on allergic diseases and the risk of tea intake. (A) allergic asthma. (B) atopic dermatitis. (C) allergic rhinitis. SNP, single‐nucleotide polymorphism.

In the bidirectional analyses conducted using the IVW method, we found no evidence of potential causal effects of genetically predicted AD, AR, and AA on tea intake. The odds ratios were 1.002 (95% CI 0.988–1.017, *p* = 0.756), 1.226 (95% CI 0.974–1.544, *p* = 0.083), and 1.011 (95% CI 0.996–1.025, *p* = 0.144), respectively (Table 2). These findings were consistent with the results obtained from the MR Egger and weighted median model. The estimated effect sizes for SNPs associated with AD, AR, and AA on tea intake were illustrated in a scatter plot (Figure 3).

**TABLE 2:** Odds ratios for the associations between genetically predicted allergic diseases and tea intake.

Exposures	Cases	Controls	nSNPs	Method	OR (95% CI)	*p*
AD	774,187	796,661	20			
				MR Egger	1.024 (0.975, 1.074)	0.355
				Weighted median	1.014 (0.996, 1.032)	0.124
				IVW	1.002 (0.988, 1.017)	0.756
				Weighted mode	1.015 (0.989, 1.041)	0.288
AR	457,183	484,598	28			
				MR Egger	0.950 (0.438, 2.060)	0.898
				Weighted median	1.136 (0.838, 1.540)	0.411
				IVW	1.226 (0.974, 1.544)	0.083
				Weighted mode	1.046 (0.598, 1.830)	0.876
AA	131,051	135,910	7			
				MR Egger	0.886 (0.734, 1.070)	0.264
				Weighted median	1.010 (0.992, 1.028)	0.298
				IVW	1.011 (0.996, 1.025)	0.144
				Weighted mode	1.007 (0.985, 1.030)	0.545

Abbreviatons: CI, confidence interval; IVW, inverse variance weighted; nSNPs, number of single‐nucleotide polymorphisms; OR, odds ratio.

### 3.3 Results for Sensitivity Analyses

The MR Egger regression intercept test and the MR‐PRESSO global test revealed no evidence of horizontal pleiotropy for SNPs associated with tea intake, with AD, AR, and AA as outcomes (all *p* > 0.05) (Table 3). The Cochran *Q* statistic indicated low heterogeneity, suggesting the reliability of these SNPs (all *p* > 0.05). We conducted a leave‐one‐out sensitivity analysis using conventional IVW methods. The results remained consistent after excluding individual SNPs in the leave‐one‐out analysis, indicating that no single SNP exerted an undue influence on the overall estimates (Figure S1). In bidirectional analyses, both the MR Egger regression intercept and MR‐PRESSO global test found no evidence of horizontal pleiotropy for SNPs associated with AD, AR, and AA, with tea intake as the outcome (all *p* > 0.05) (Table 4). Similarly, no heterogeneity was detected in the Cochran *Q* statistic (all *p* > 0.05) (Table 4). The leave‐one‐out analysis also suggested that no single SNP had an undue influence on the overall estimates (Figure S2).

**TABLE 3:** Pleiotropy and heterogeneity analyses for the association of tea intake with AD, AR, and AA.

Outcomes	nSNPs	Cochrane's *Q* test		MR Egger pleiotropy test			MR‐PRESSO	*p*
		*Q*	*p*	Intercept	SE	*p*		
AD	37	42.473	0.212	0.0012	0.0073	0.871	44.688	0.238
AR	39	33.760	0.431	−0.0002	0.0002	0.315	35.508	0.444
AA	40	46.669	0.186	−0.0012	0.0093	0.900	48.976	0.206

Abbreviations: nSNPs, number of single‐nucleotide polymorphisms; SE, standard error.

**TABLE 4:** Pleiotropy and heterogeneity analyses for the association of allergic diseases with tea intake.

Exposures	nSNPs	Cochrane's *Q* test		MR Egger pleiotropy test			MR‐PRESSO	*p*
		*Q*	*p*	Intercept	SE	*p*		
AD	20	26.379	0.120	−0.002	0.002	0.382	29.201	0.120
AR	28	11.002	0.997	0.001	0.002	0.505	0.761	1.000
AA	7	4.928	0.553	0.018	0.013	0.229	6.530	0.589

Abbreviations: nSNPs, number of single‐nucleotide polymorphisms; SE, standard error.

## 4 Discussion

To our knowledge, this is the first bidirectional MR study that probes the potential causal relationship between tea intake with AD, AR, and AA. The MR analysis in our study suggests that an increase in tea intake could potentially lead to a reduction in the risk of AD and AA, a finding that aligns with our initial hypothesis. However, we failed to discover a significant causal relationship between tea intake and AR. Furthermore, the bidirectional studies did not yield evidence to endorse that genetically inferred AD, AR, or AA had a causal association with tea intake.

Tea, as a natural herbal beverage, exerts antiallergic effects not only in allergy prevention, but also in allergy treatment. It has been proved that tea has the antiallergic ability against food allergy, respiratory allergy, atopic dermatitis, and anaphylaxis (Li et al. 2021). Typically, the reduction in the ratios of IgE and histamine are primary indicators used to assess the antiallergic efficacy of specific compounds, including polyphenols, saponins, and polysaccharides. It is hypothesized that these compounds in tea interfere with critical allergy phases by inhibiting the formation of the allergen‐IgE complex and reducing cytokine release from Th2 cells (Mfengu et al. 2021). Tea saponins, which are natural nonionic surfactants, can be found in various parts of the tea plant, including the leaves, stem, flowers, and seeds (Guo et al. 2018; Yu and He 2018). Research has demonstrated that saponins possess antiallergic properties, showing potential against conditions such as AD and AA (Choi, Bae, et al. 2014; Choi, Jin, et al. 2014; Sipos et al. 2013). These findings suggest that tea extract could potentially be a viable treatment option for allergic diseases.

Allergic rhinitis is a chronic, IgE‐mediated inflammatory disorder affecting the respiratory tract. A formulation of black cumin, licorice, anise, and tea (denoted BLAB tea) is traditionally used to relief allergy reaction including allergic rhinitis. Liao et al. revealed that the aqueous extract of BLAB tea mitigates AR by inhibiting the release of Th2 cytokines and histamine in the nasal mucosa and serum of the mice (Liao et al. 2021). Epigallocatechin gallate (EGCG) is an active catechin in tea and has multiple biological functions, such as anti‐inflammation and immune regulation (Khan et al. 2006). Previous study demonstrated that EGCG could potentially alleviate AR by suppressing the release of IgE and histamine in ovalbumin (OVA)‐induced AR model mice (Fu, Fu, et al. 2017; Fu, Li, et al. 2017). However, our MR study did not establish a causal relationship between tea intake and AR. Several factors could explain this: Firstly, BLAB tea is a composite preparation where tea merely serves as an adjuvant and may not be the primary component in mitigating AR. Secondly, although EGCG is a constituent of tea, the concentration of the extract employed in the study significantly exceeds the concentration and intake of daily tea. Furthermore, the research findings are based on OVA‐induced AR model mice. The AR development mechanism in patients might differ from this model. Additional clinical observational studies are required to substantiate the correlation between tea intake and AR.

Atopic dermatitis is a chronic skin condition characterized by recurrent episodes of severe itching. The primary symptom of AD is itchy eczema. Epidermal keratinocytes, essential for skin barrier function, synthesize a protein called filaggrin, and mutations in the filaggrin gene significantly elevate the risk of developing AD (Palmer et al. 2006). In a randomized controlled trial (RCT) study, Abe et al. discovered that tea intake could potentially serve as a safe and effective alternative to existing therapeutic approaches for AD (Abe et al. 2022). Our MR analysis further revealed a correlation between an increase in genetically predicted tea intake and a lower risk of AD, corroborating the results of the RCT study. Promoting the consumption of tea as a potential natural therapeutic strategy against AD could have significant implications for public health.

Allergic asthma is a chronic inflammatory disease of the lungs, typically marked by airway obstruction, wheezing, and heightened airway responsiveness. It is characterized by an exaggerated response to allergens, leading to airway edema and increased mucus secretion. Choi et al. observed that EGCG diminishes mucin expression in both the asthma‐model mouse and the nasal epithelial cells of patients suffering from allergic inflammation (Choi, Bae, et al. 2014). A recent study also demonstrates that EGCG treatment relieves asthmatic symptoms in mice by suppressing HIF‐1α/VEGFA‐mediated M2 skewing of macrophages (Yang and Li 2022). In addition, our results show that tea intake was causally associated with the risk of AA, which is consistent with the findings.

The strength of our study lies in examining the effects of tea intake on allergic diseases through genetic analysis, using data from GWAS sources. To meet the assumptions of MR studies, it is essential to check for horizontal pleiotropy and heterogeneity adequately. The MR‐PRESSO global test was used to identify outliers and demonstrate pleiotropy. The robustness of our significant findings was further validated by excluding heterogeneity using Cochrane's *Q* test and leave‐one‐out sensitivity analysis.

Undoubtedly, our study has several limitations. First, the examined GWASs were primarily conducted in individuals of European ancestry, limiting our findings' generalizability to other ethnicities such as East Asians. Second, the brewing method and type of tea were unclear because there was no specific information about them. The effects of tea type and brewing methods were not assessed. Finally, the data used in this study are summary statistics data, so there is no individual information, and it is impossible to accurately calculate the sample overlap between exposure and outcome.

## 5 Conclusion

This is the first MR study to investigate the causal association between tea intake and allergic diseases (including AD, AR, and AA). Using genetic data, our MR analysis suggested that increased tea intake may reduce the risk of AD and AA. This suggests that tea intake is likely a trigger or a prevention strategy for AD and AA.

## Author Contributions

**Jinjin Zhang:** conceptualization (lead), data curation (lead), writing – original draft (lead). **Yuhan Liu:** data curation (equal), software (equal). **Jiawei Zhang:** formal analysis (equal), validation (equal). **Fei Zeng:** funding acquisition (equal), software (equal). **Yuqing Wu:** software (equal). **Xuexue Zhang:** visualization (equal). **Daying Zhang:** conceptualization (equal), supervision (equal), writing – review and editing (equal). **Mengye Zhu:** conceptualization (equal), funding acquisition (equal), writing – review and editing (lead).

## Conflicts of Interest

The authors declare no conflicts of interest.

## Supporting information


**Table S1.** The detailed information of the instrumental variables in each trait.
**Table S2.** Data sources used to identify genetic variants in this study.
**Figure S1.** MR leave‐one‐out sensitivity analyses for tea intake on allergic diseases.
**Figure S2.** MR leave‐one‐out sensitivity analyses for allergic diseases on tea intake.

## Data Availability

All the GWAS data employed in this research can be accessed through the IEU Open GWAS project, which is available at (https://gwas.mrcieu.ac.uk/).
