# Supplementary material for: Exploring Genetic Association of Tea Intake With Allergic Diseases Among European Population: A Bidirectional Mendelian Randomization Study
Source: Food Sci Nutr. 2024 Nov 1;12(12):10223–30. doi: 10.1002/fsn3.4574 (PMC11666899; doi:10.1002/fsn3.4574)

Supplementary Material

**1. Supplementary Tables**

**Table S1.** The detailed information of the instrumental variables in each trait.

**Table S2.** Data sources used to identify genetic variants in this study.

**2. Supplementary Figures**

**Figure S1.** MR leave-one-out sensitivity analyses for tea intake on allergic diseases.

**Figure S2.** MR leave-one-out sensitivity analyses for allergic diseases on tea intake.

1. **Supplementary Tables**

| **Table S1.** The detailed information of the instrumental variables in each trait. | | | | | | | | | | | |
| --- | --- | --- | --- | --- | --- | --- | --- | --- | --- | --- | --- |
| Traits | SNP | Chr | Pos. | Effect allele | Other allele | EAF | Beta | SE | *P* value | R^2^ | F statistic |
| Tea intake | rs11587444 | 1 | 150722844 | G | A | 0.393 | 0.014 | 0.002 | 1.00E-10 | 9.40E-05 | 41.789 |
| Tea intake | rs56188862 | 1 | 174189269 | C | T | 0.387 | -0.016 | 0.002 | 4.30E-13 | 1.18E-04 | 52.497 |
| Tea intake | rs962242 | 1 | 154592140 | C | T | 0.225 | 0.014 | 0.003 | 1.20E-08 | 7.30E-05 | 32.474 |
| Tea intake | rs1156588 | 2 | 58515375 | G | A | 0.210 | -0.015 | 0.003 | 2.90E-09 | 7.93E-05 | 35.241 |
| Tea intake | rs57462170 | 3 | 50239803 | A | G | 0.109 | 0.019 | 0.003 | 1.90E-08 | 7.11E-05 | 31.620 |
| Tea intake | rs2117137 | 3 | 89525505 | G | A | 0.405 | 0.013 | 0.002 | 1.70E-09 | 8.14E-05 | 36.338 |
| Tea intake | rs1481012 | 4 | 89039082 | G | A | 0.112 | -0.026 | 0.003 | 5.30E-15 | 1.37E-04 | 61.148 |
| Tea intake | rs72797284 | 5 | 152031650 | G | A | 0.271 | -0.017 | 0.002 | 7.00E-13 | 1.16E-04 | 51.558 |
| Tea intake | rs34619 | 5 | 60465365 | A | G | 0.431 | 0.012 | 0.002 | 4.30E-08 | 6.73E-05 | 30.021 |
| Tea intake | rs7757102 | 6 | 137222671 | G | A | 0.555 | -0.012 | 0.002 | 3.10E-08 | 6.88E-05 | 30.624 |
| Tea intake | rs2478875 | 6 | 51283110 | G | A | 0.209 | 0.022 | 0.003 | 5.10E-17 | 1.58E-04 | 70.299 |
| Tea intake | rs149805207 | 6 | 137095269 | G | A | 0.009 | -0.072 | 0.013 | 1.10E-08 | 8.76E-05 | 32.685 |
| Tea intake | rs11768350 | 7 | 17561651 | C | T | 0.159 | -0.021 | 0.003 | 1.20E-12 | 1.14E-04 | 50.502 |
| Tea intake | rs4410790 | 7 | 17284577 | C | T | 0.631 | 0.041 | 0.002 | 3.40E-76 | 7.66E-04 | 341.270 |
| Tea intake | rs1078032 | 7 | 17464410 | C | T | 0.436 | 0.012 | 0.002 | 3.20E-08 | 6.95E-05 | 30.579 |
| Tea intake | rs9648476 | 7 | 39293033 | A | G | 0.623 | 0.013 | 0.002 | 1.10E-08 | 7.34E-05 | 32.722 |
| Tea intake | rs10273455 | 7 | 17762666 | A | C | 0.552 | -0.012 | 0.002 | 4.60E-08 | 6.74E-05 | 29.865 |
| Tea intake | rs17685 | 7 | 75616105 | A | G | 0.278 | 0.023 | 0.002 | 1.60E-22 | 2.13E-04 | 95.364 |
| Tea intake | rs13282783 | 8 | 22088975 | T | C | 0.286 | -0.014 | 0.002 | 7.90E-09 | 7.53E-05 | 33.289 |
| Tea intake | rs10764990 | 10 | 129152608 | A | G | 0.607 | -0.012 | 0.002 | 1.90E-08 | 7.09E-05 | 31.589 |
| Tea intake | rs2351187 | 10 | 86850616 | A | G | 0.319 | 0.013 | 0.002 | 1.60E-08 | 7.23E-05 | 31.959 |
| Tea intake | rs10752269 | 10 | 12692902 | A | G | 0.506 | -0.013 | 0.002 | 1.30E-09 | 8.28E-05 | 36.878 |
| Tea intake | rs4418728 | 10 | 94839724 | T | G | 0.451 | -0.012 | 0.002 | 3.70E-08 | 6.79E-05 | 30.311 |
| Tea intake | rs10741694 | 11 | 16286183 | C | T | 0.628 | 0.015 | 0.002 | 7.90E-12 | 1.05E-04 | 46.784 |
| Tea intake | rs11022752 | 11 | 13307622 | G | A | 0.269 | 0.013 | 0.002 | 2.50E-08 | 7.02E-05 | 31.100 |
| Tea intake | rs17245213 | 11 | 1679769 | A | G | 0.208 | -0.015 | 0.003 | 2.00E-08 | 7.07E-05 | 31.521 |
| Tea intake | rs977474 | 12 | 11284772 | T | C | 0.834 | 0.022 | 0.003 | 2.40E-14 | 1.32E-04 | 58.180 |
| Tea intake | rs2645929 | 13 | 56444529 | G | A | 0.813 | -0.015 | 0.003 | 3.50E-08 | 6.83E-05 | 30.424 |
| Tea intake | rs6829 | 13 | 111531264 | T | C | 0.596 | -0.012 | 0.002 | 3.70E-08 | 6.84E-05 | 30.282 |
| Tea intake | rs17576658 | 13 | 100272019 | A | G | 0.247 | -0.013 | 0.002 | 4.10E-08 | 6.76E-05 | 30.117 |
| Tea intake | rs7999399 | 13 | 89233505 | T | C | 0.556 | 0.012 | 0.002 | 4.00E-08 | 6.77E-05 | 30.127 |
| Tea intake | rs2472297 | 15 | 75027880 | T | C | 0.262 | 0.053 | 0.002 | 2.30E-109 | 1.10E-03 | 493.646 |
| Tea intake | rs12591786 | 15 | 60902512 | T | C | 0.159 | -0.018 | 0.003 | 3.70E-10 | 9.08E-05 | 39.274 |
| Tea intake | rs9937354 | 16 | 53799847 | A | G | 0.424 | -0.014 | 0.002 | 4.90E-11 | 9.70E-05 | 43.231 |
| Tea intake | rs512404 | 16 | 63031551 | T | G | 0.225 | 0.015 | 0.003 | 4.50E-09 | 7.86E-05 | 34.413 |
| Tea intake | rs2279844 | 17 | 40819809 | A | G | 0.379 | -0.012 | 0.002 | 4.00E-08 | 6.77E-05 | 30.151 |
| Tea intake | rs4808193 | 19 | 19410622 | C | T | 0.335 | 0.015 | 0.002 | 1.70E-11 | 1.02E-04 | 45.240 |
| Tea intake | rs57631352 | 19 | 4338173 | G | A | 0.297 | -0.013 | 0.002 | 1.70E-08 | 7.17E-05 | 31.868 |
| Tea intake | rs4817505 | 21 | 34343828 | C | T | 0.390 | 0.015 | 0.002 | 4.20E-12 | 1.08E-04 | 48.012 |
| Tea intake | rs9624470 | 22 | 24820268 | A | G | 0.580 | 0.025 | 0.002 | 1.30E-31 | 3.10E-04 | 136.840 |
| Atopic dermatitis | rs12126142 | 1 | 154425456 | A | G | 0.362 | 0.063 | 0.011 | 6.21E-09 | 0.002 | 33.597 |
| Atopic dermatitis | rs17371133 | 1 | 172828480 | C | A | 0.483 | 0.065 | 0.010 | 2.07E-10 | 0.002 | 40.734 |
| Atopic dermatitis | rs12123821 | 1 | 152179152 | T | C | 0.045 | 0.270 | 0.025 | 2.2E-26 | 0.006 | 113.330 |
| Atopic dermatitis | rs13343107 | 1 | 173168321 | G | A | 0.237 | 0.075 | 0.012 | 5.11E-10 | 0.002 | 38.830 |
| Atopic dermatitis | rs1861246 | 2 | 102966783 | C | T | 0.783 | -0.113 | 0.012 | 1.51E-20 | 0.004 | 86.399 |
| Atopic dermatitis | rs7699742 | 4 | 123264485 | C | T | 0.378 | 0.060 | 0.010 | 5.45E-09 | 0.002 | 34.274 |
| Atopic dermatitis | rs847 | 5 | 131996669 | C | T | 0.739 | -0.093 | 0.011 | 3.75E-17 | 0.003 | 70.651 |
| Atopic dermatitis | rs28383323 | 6 | 32594039 | A | G | 0.238 | -0.119 | 0.016 | 1.1E-13 | 0.005 | 55.131 |
| Atopic dermatitis | rs11786685 | 8 | 81275835 | G | A | 0.661 | -0.072 | 0.011 | 1.99E-11 | 0.002 | 44.568 |
| Atopic dermatitis | rs6996614 | 8 | 126609868 | A | C | 0.449 | 0.081 | 0.014 | 1.55E-08 | 0.003 | 32.138 |
| Atopic dermatitis | rs479844 | 11 | 65551957 | G | A | 0.573 | 0.072 | 0.010 | 2.41E-12 | 0.003 | 48.729 |
| Atopic dermatitis | rs11033603 | 11 | 36434542 | A | G | 0.042 | 0.165 | 0.023 | 1.33E-12 | 0.002 | 50.275 |
| Atopic dermatitis | rs61893488 | 11 | 76349107 | A | G | 0.086 | -0.117 | 0.018 | 2.91E-11 | 0.002 | 44.117 |
| Atopic dermatitis | rs7936070 | 11 | 76293527 | T | G | 0.452 | 0.091 | 0.010 | 7.19E-19 | 0.004 | 78.722 |
| Atopic dermatitis | rs3947727 | 12 | 68652099 | T | C | 0.599 | 0.071 | 0.011 | 1.09E-11 | 0.002 | 46.240 |
| Atopic dermatitis | rs12586305 | 14 | 35654467 | G | T | 0.159 | 0.091 | 0.014 | 7.92E-11 | 0.002 | 42.250 |
| Atopic dermatitis | rs3862469 | 16 | 11194080 | T | C | 0.313 | -0.076 | 0.011 | 3.59E-12 | 0.002 | 48.488 |
| Atopic dermatitis | rs35073649 | 17 | 47422510 | T | C | 0.393 | 0.079 | 0.010 | 2.04E-14 | 0.003 | 58.085 |
| Atopic dermatitis | rs2918302 | 19 | 8785744 | A | G | 0.174 | 0.092 | 0.013 | 5.1E-12 | 0.002 | 47.330 |
| Atopic dermatitis | rs3848669 | 20 | 62300811 | T | G | 0.779 | 0.101 | 0.013 | 2.84E-15 | 0.004 | 62.139 |
| Atopic dermatitis | rs2075943 | 22 | 37319009 | A | G | 0.576 | 0.065 | 0.011 | 2.09E-09 | 0.002 | 36.000 |
| Allergic rhinitis | rs2988277 | 1 | 167431352 | T | C | 0.388 | -0.003 | 0.000 | 6.00E-12 | 5.23E-06 | 46.954 |
| Allergic rhinitis | rs2070901 | 1 | 161185058 | T | G | 0.277 | 0.003 | 0.001 | 4.90E-09 | 3.80E-06 | 34.276 |
| Allergic rhinitis | rs9807989 | 2 | 102971200 | C | T | 0.326 | -0.005 | 0.001 | 5.60E-23 | 1.14E-05 | 96.095 |
| Allergic rhinitis | rs891058 | 2 | 8442547 | A | G | 0.287 | -0.004 | 0.001 | 1.10E-13 | 6.07E-06 | 54.766 |
| Allergic rhinitis | rs149401825 | 2 | 198491341 | T | C | 0.503 | -0.003 | 0.000 | 7.80E-11 | 4.71E-06 | 41.053 |
| Allergic rhinitis | rs34290285 | 2 | 242698640 | A | G | 0.257 | -0.005 | 0.001 | 5.60E-20 | 9.21E-06 | 83.484 |
| Allergic rhinitis | rs763342 | 3 | 187639375 | C | A | 0.339 | 0.003 | 0.000 | 1.80E-08 | 3.41E-06 | 31.072 |
| Allergic rhinitis | rs3856972 | 4 | 4768913 | G | A | 0.385 | 0.003 | 0.000 | 2.20E-09 | 4.02E-06 | 35.919 |
| Allergic rhinitis | rs7688384 | 4 | 123229132 | T | C | 0.318 | -0.004 | 0.001 | 6.30E-15 | 6.77E-06 | 60.890 |
| Allergic rhinitis | rs7735355 | 5 | 110153295 | C | A | 0.160 | 0.006 | 0.001 | 5.20E-22 | 1.06E-05 | 93.033 |
| Allergic rhinitis | rs1438673 | 5 | 110467499 | T | C | 0.498 | -0.004 | 0.000 | 5.90E-21 | 9.91E-06 | 88.285 |
| Allergic rhinitis | rs34463936 | 5 | 35850149 | T | C | 0.276 | -0.005 | 0.001 | 4.70E-20 | 9.36E-06 | 84.742 |
| Allergic rhinitis | rs62390337 | 5 | 159914884 | T | C | 0.240 | -0.003 | 0.001 | 2.40E-08 | 3.51E-06 | 31.577 |
| Allergic rhinitis | rs202120978 | 6 | 33028728 | C | A | 0.158 | 0.004 | 0.001 | 8.10E-11 | 5.34E-06 | 42.131 |
| Allergic rhinitis | rs147563297 | 6 | 32237550 | A | G | 0.012 | 0.013 | 0.002 | 7.80E-10 | 4.13E-06 | 37.222 |
| Allergic rhinitis | rs28359896 | 6 | 32606547 | A | G | 0.579 | -0.005 | 0.000 | 4.10E-28 | 1.45E-05 | 121.036 |
| Allergic rhinitis | rs9275075 | 6 | 32648550 | A | G | 0.122 | 0.007 | 0.001 | 9.20E-19 | 1.08E-05 | 77.941 |
| Allergic rhinitis | rs71540435 | 6 | 32547073 | G | A | 0.066 | 0.006 | 0.001 | 1.30E-08 | 4.43E-06 | 32.312 |
| Allergic rhinitis | rs111699363 | 7 | 20445945 | A | G | 0.028 | 0.008 | 0.001 | 3.20E-08 | 3.46E-06 | 30.965 |
| Allergic rhinitis | rs1888909 | 9 | 6197392 | C | T | 0.740 | -0.005 | 0.001 | 1.40E-20 | 9.60E-06 | 86.469 |
| Allergic rhinitis | rs2197415 | 10 | 9062856 | G | T | 0.583 | 0.003 | 0.000 | 4.60E-11 | 4.70E-06 | 42.378 |
| Allergic rhinitis | rs34415530 | 12 | 56444632 | T | C | 0.335 | 0.003 | 0.000 | 5.50E-09 | 3.75E-06 | 33.847 |
| Allergic rhinitis | rs56375023 | 15 | 67448363 | A | G | 0.230 | 0.004 | 0.001 | 1.20E-13 | 6.18E-06 | 55.763 |
| Allergic rhinitis | rs2074585 | 15 | 91009484 | A | G | 0.527 | -0.003 | 0.000 | 1.60E-09 | 4.06E-06 | 36.224 |
| Allergic rhinitis | rs34753162 | 15 | 61069177 | C | T | 0.141 | -0.004 | 0.001 | 5.80E-11 | 4.77E-06 | 42.398 |
| Allergic rhinitis | rs11076469 | 16 | 49210789 | T | G | 0.236 | -0.003 | 0.001 | 9.90E-10 | 4.13E-06 | 37.378 |
| Allergic rhinitis | rs3816470 | 17 | 37985801 | G | A | 0.539 | -0.003 | 0.000 | 6.90E-10 | 4.25E-06 | 38.389 |
| Allergic rhinitis | rs34210653 | 17 | 4535314 | A | G | 0.020 | -0.011 | 0.002 | 2.10E-10 | 4.53E-06 | 41.138 |
| Allergic rhinitis | rs9889953 | 17 | 38754800 | A | G | 0.653 | 0.003 | 0.000 | 1.20E-09 | 4.11E-06 | 37.239 |
| Allergic rhinitis | rs6094570 | 20 | 45682341 | G | A | 0.259 | -0.004 | 0.001 | 2.10E-11 | 4.94E-06 | 44.253 |
| Allergic asthma | rs62192043 | 2 | 242711282 | A | G | 0.235 | -0.144 | 0.026 | 2.49E-08 | 0.007 | 31.109 |
| Allergic asthma | rs79881201 | 5 | 110427795 | T | C | 0.309 | 0.138 | 0.023 | 2.64E-09 | 0.008 | 35.485 |
| Allergic asthma | rs28694082 | 5 | 129099176 | T | C | 0.234 | 0.139 | 0.025 | 4.40E-08 | 0.007 | 29.861 |
| Allergic asthma | rs847 | 5 | 131996669 | C | T | 0.637 | -0.131 | 0.022 | 4.15E-09 | 0.008 | 34.667 |
| Allergic asthma | rs7110818 | 11 | 76292575 | T | C | 0.405 | 0.125 | 0.022 | 8.40E-09 | 0.008 | 33.341 |
| Allergic asthma | rs74630264 | 16 | 27316975 | A | G | 0.082 | -0.246 | 0.040 | 5.05E-10 | 0.009 | 38.723 |
| Allergic asthma | rs8074437 | 17 | 38076137 | G | T | 0.444 | 0.133 | 0.022 | 7.59E-10 | 0.009 | 37.971 |

| **Table S2.** Data sources used to identify genetic variants in this study. | | | | | | |
| --- | --- | --- | --- | --- | --- | --- |
| **Phenotypes** | **Cases** | **Controls** | **Sample size** | **Population** | **Year** | **Date source** |
| Tea intake | - | - | 447,485 | European | 2018 | MRC-IEU |
| Atopic dermatitis | 22,474 | 774,187 | 796,661 | European | 2021 | Sliz E et al.^16^ |
| Allergic rhinitis | 27,415 | 457,183 | 484,598 | European | 2021 | Dönertaş HM et al.^17^ |
| Allergic asthma | 4859 | 131,051 | 135,910 | European | 2021 | FinnGen |

1. **Supplementary Figures**

**Figure S1.** MR leave-one-out sensitivity analyses for tea intake on allergic diseases. (A) Atopic dermatitis, (B) Allergic rhinitis, and (C) Allergic asthma.


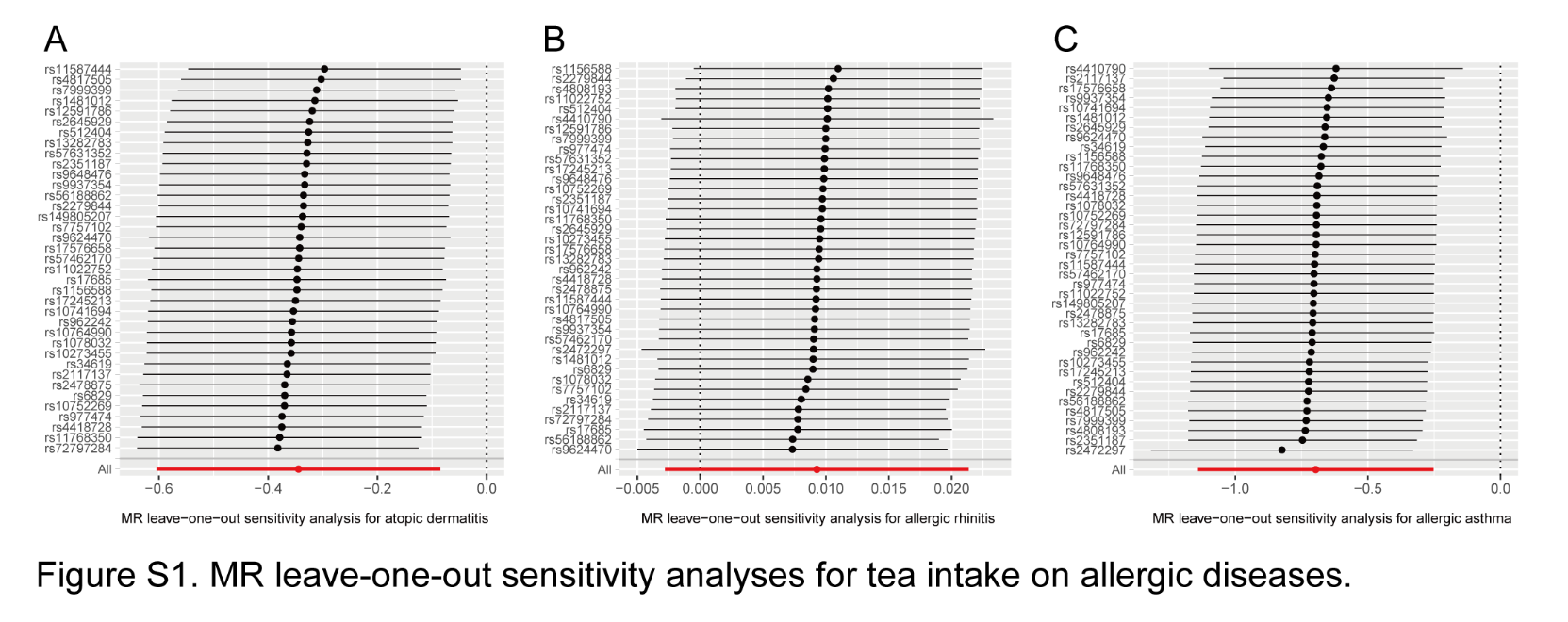


**Figure S2.** MR leave-one-out sensitivity analyses for allergic diseases on tea intake. (A) Atopic dermatitis, (B) Allergic rhinitis, and (C) Allergic asthma.


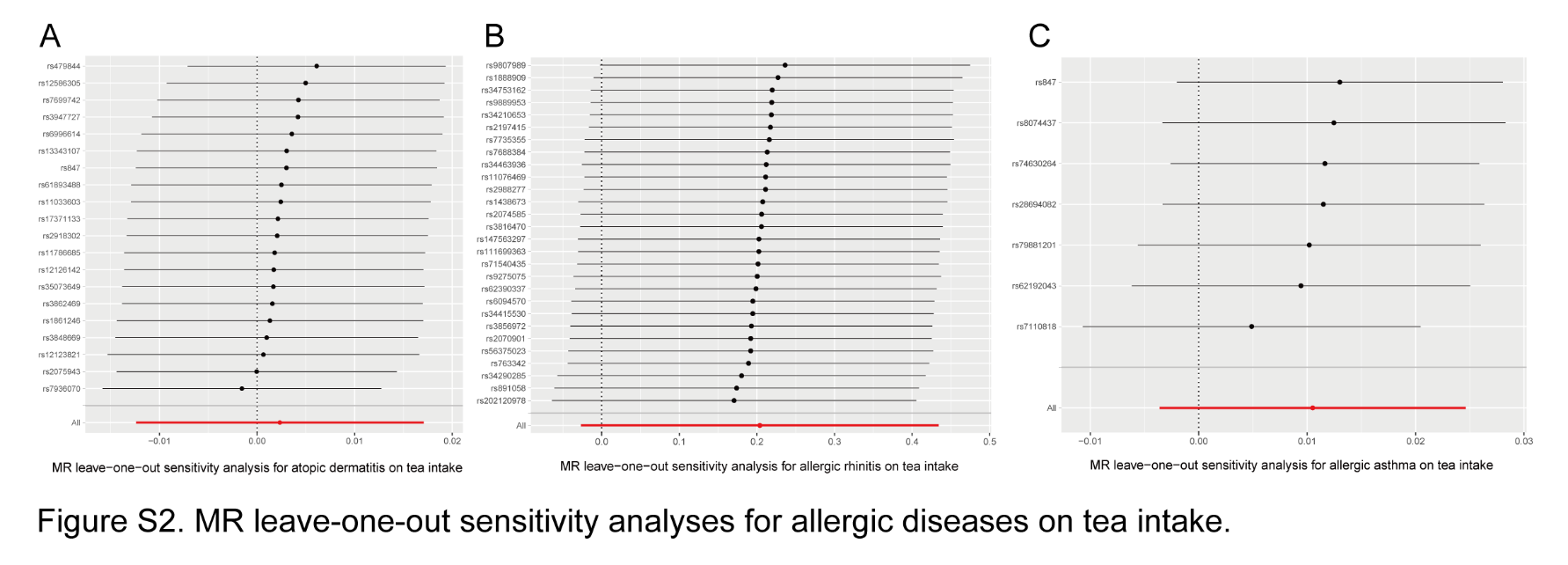

Supplement: Supplementary file 1 — Table S1. The detailed information of the instrumental variables in each trait. Table S2. Data sources used to identify genetic variants in this study. Figure S1. MR leave‐one‐out sensitivity analyses for tea intake on allergic diseases. Figure S2. MR leave‐one‐out sensitivity analyses for allergic diseases on tea intake. [file FSN3-12-10223-s001.docx]
